# Fenton Reaction Induced Cancer in Wild Type Rats Recapitulates Genomic Alterations Observed in Human Cancer

**DOI:** 10.1371/journal.pone.0043403

**Published:** 2012-08-29

**Authors:** Shinya Akatsuka, Yoriko Yamashita, Hiroki Ohara, Yu-Ting Liu, Masashi Izumiya, Koichiro Abe, Masako Ochiai, Li Jiang, Hirotaka Nagai, Yasumasa Okazaki, Hideki Murakami, Yoshitaka Sekido, Eri Arai, Yae Kanai, Okio Hino, Takashi Takahashi, Hitoshi Nakagama, Shinya Toyokuni

**Affiliations:** 1 Departments of Pathology and Biological Responses, Nagoya University Graduate School of Medicine, Showa-ku, Nagoya, Japan; 2 Department of Pathology and Biology of Diseases, Kyoto University Graduate School of Medicine, Sakyo-ku, Kyoto, Japan; 3 Division of Cancer Development System, National Cancer Center Research Institute, Chuo-ku, Tokyo, Japan; 4 Department of Internal Medicine, Teikyo University School of Medicine, Itabashi-ku, Tokyo, Japan; 5 Division of Molecular Oncology, Aichi Cancer Center Research Institute, Chikusa-Ku, Nagoya, Japan; 6 Division of Molecular Pathology, National Cancer Center Research Institute, Chuo-ku, Tokyo, Japan; 7 Department of Pathology and Oncology, Juntendo University School of Medicine, Bunkyo-ku, Tokyo, Japan; 8 Molecular Carcinogenesis, Nagoya University Graduate School of Medicine, Showa-ku, Nagoya, Japan; Texas Tech University, United States of America

## Abstract

Iron overload has been associated with carcinogenesis in humans. Intraperitoneal administration of ferric nitrilotriacetate initiates a Fenton reaction in renal proximal tubules of rodents that ultimately leads to a high incidence of renal cell carcinoma (RCC) after repeated treatments. We performed high-resolution microarray comparative genomic hybridization to identify characteristics in the genomic profiles of this oxidative stress-induced rat RCCs. The results revealed extensive large-scale genomic alterations with a preference for deletions. Deletions and amplifications were numerous and sometimes fragmented, demonstrating that a Fenton reaction is a cause of such genomic alterations *in vivo*. Frequency plotting indicated that two of the most commonly altered loci corresponded to a *Cdkn2a*/*2b* deletion and a *Met* amplification. Tumor sizes were proportionally associated with *Met* expression and/or amplification, and clustering analysis confirmed our results. Furthermore, we developed a procedure to compare whole genomic patterns of the copy number alterations among different species based on chromosomal syntenic relationship. Patterns of the rat RCCs showed the strongest similarity to the human RCCs among five types of human cancers, followed by human malignant mesothelioma, an iron overload-associated cancer. Therefore, an iron-dependent Fenton chemical reaction causes large-scale genomic alterations during carcinogenesis, which may result in distinct genomic profiles. Based on the characteristics of extensive genome alterations in human cancer, our results suggest that this chemical reaction may play a major role during human carcinogenesis.

## Introduction

Cancer is a disease of accumulated genomic alterations, presumably caused by a systematic process during cellular injury and repair. Causative agents for carcinogenesis are numerous including γ-radiation, ultraviolet radiation, inflammation, chemicals and iron overload [1]. Genomic data of a variety of human cancers is currently analyzed either with array-based comparative genomic hybridization (CGH) [2] or next-generation sequencing [3], [4]. These projects are performed to find causative gene mutations that will lead to identifying novel chemicals or antibodies directed for the interactions of responsible signaling molecules. These efforts are expected to result in developments of effective drugs. However, cancer prevention in daily life is as important as its therapy.

In the present study, we sought to resolve roles of iron-mediated oxidative stress during carcinogenesis using array-based CGH. Oxidative stress is constitutively caused by the metabolism of molecular oxygen [5], but is mainly regulated by various antioxidant systems. However, in a variety of pathological conditions, oxidative stress loads exceed the antioxidant capacity [6]. Iron is the most abundant heavy metal in mammals, such as rodents and humans. Whereas iron is essential for oxygen transport as a component of hemoglobin, excess iron has been associated with carcinogenesis [7], [8], presumably through a Fenton reaction [9]. Ionic forms of iron are barely soluble at a neutral pH, but ferric nitrilotriacetate (Fe-NTA), an iron chelate, is soluble at pH 7.4 and is an efficient catalytic agent for the Fenton reaction [10]. In the 1980s, our group established that repeated intraperitoneal administrations of Fe-NTA induce a high incidence of renal cell carcinoma (RCC) in rodents [11], [12]. Later, we showed that the renal injury occurs through a Fenton reaction with a variety of hydroxyl radical-mediated chemical products, such as 8-hydroxy-2′-deoxyguanosine [13], [14] and 4-hydroxy-nonenal [15], [16]. It is established that an iron overload in many pathological conditions is associated with the presence of catalytic iron [17], [18].

Accordingly, by evaluating whole genome of RCCs, we could find a general principle for the genomic alterations under oxidatively-stressed conditions. We reported a *Cdkn2a/2b* deletion using microsatellite analysis in this model [19]. In this study, we evaluated the whole genome of Fe-NTA-induced rat RCCs and their cell lines using array-based CGHs. Furthermore, we transformed the data into a human genome through chromosomal syntenic relationship and analyzed the association.

## Results

### Genome-wide Views of DNA Copy Number Alterations in Fe-NTA-induced Rat RCCs

Fifteen rat RCC DNA samples, which included 13 primary tumor samples and 2 cell line samples, were hybridized on Agilent oligonucleotide microarrays for CGH with 181,978 genomic loci (GEO accession: GSE36101). Comparing different array-based CGH profiles in a quantitative manner is difficult. A shift in the mean copy number is caused by polyploidy and the contamination of normal cells. Therefore, we have developed a statistical method that considers these factors to estimate the chromosomal copy number (**Methods S1**). In this paper, array-based CGH profile data analyses are based on the estimated copy numbers using this method.

**Figure 1:**
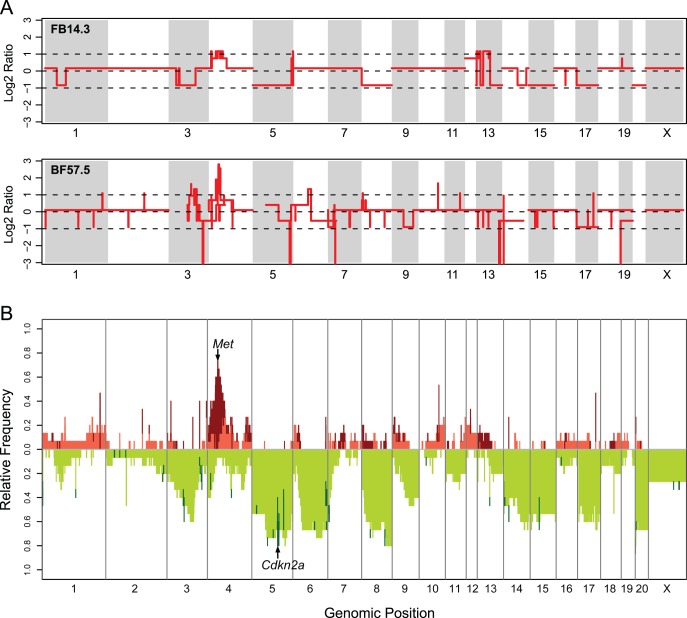
Genome-wide views of DNA copy number alterations in Fe-NTA induced rat renal cell carcinomas (RCCs). (A) Representative array-based CGH profiles from two RCC tumors. The red lines show log2 ratios of the estimated copy number over the inferred cancer ploidy versus the genomic position for all of the CGH microarray probes. (B) Frequency distribution of copy number aberrations across the whole rat genome. The relative frequencies of amplification (dark red), gain (tomato), loss (green yellow) and homozygous deletion (dark green) within 13 RCC tumors and two RCC cell lines are plotted at each genomic position. Two cancer-related loci, *Met* and *Cdkn2a*, which were most frequently affected by copy number aberration are indicated by the arrows.

Array-based CGH profiling revealed that genomes of the Fe-NTA-induced rat RCCs are often complex and have many extensive chromosomal alterations (**Figs. 1A and S** **1**). A whole genome frequency analysis with 15 samples identified recurrent regions of a copy number aberration in the Fe-NTA-induced RCCs (**Fig. 1B**). Copy number aberrations were determined based on the distribution of the log2 ratio values that were recalculated with the estimated copy number for a set of 13 primary tumors and 2 cell lines (**Fig. S2**). In this distribution, the thresholds that represented gain and loss were chosen at ±0.377. A threshold representing amplification was chosen at +0.811 whereas a homozygous deletion (complete loss) was assigned to the position at which the copy number was estimated as 0. The most characteristic global feature uncovered by the frequency analysis was a predisposition to lose an extensively wide region of chromosomes, especially for chromosomes 3, 5, 6, 8, 9, 14, 15, 17 and 20. The second feature was a frequent amplification over a long pericentromeric region in chromosome 4.

### Frequent Chromosomal Loss in Rat Chromosome 5 and Homozygous Deletion at the *Cdkn2a/2b* Locus

**Figure 2:**
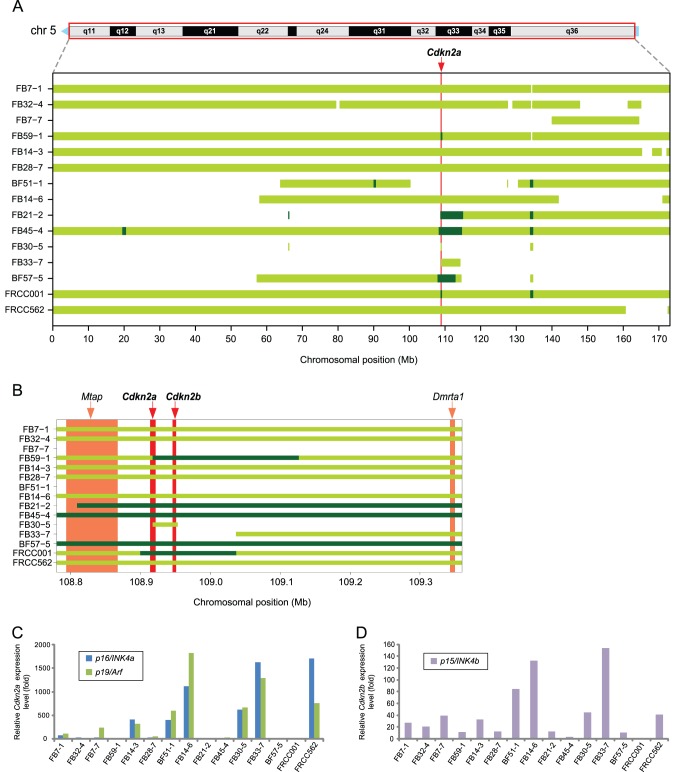
Frequent extensive chromosomal losses in rat chromosome 5, and homozygous deletions at the region including the *Cdkn2a* and *Cdkn2b* loci. (A) The bar chart represents the regions of chromosomal loss (green yellow) and homozygous deletion (dark green) along chromosome 5 for 13 RCC tumors and two RCC cell lines. The vertical red line on the background indicates the position of the *Cdkn2a* locus. (B) Magnified view of the bar chart centered on the *Cdkn2a/2b* loci. The genomic regions of all of the RefSeq genes included in the displayed range of the chromosome are depicted as vertical bars on the background. (C) Expression analysis of *Cdkn2a* (*p16^Ink4a^* and *p19^Arf^*) for 13 RCC tumors and two RCC cell lines, using real-time PCR with specific primer pairs for each different transcript. The values on the *y*-axis indicate relative mRNA expression level compared to an average of those in normal kidneys of three control rats. (D) Expression analysis of *Cdkn2b* (*p15^Ink4b^*) for 13 RCC tumors and two RCC cell lines by real-time PCR. The values on the *y*-axis indicate relative mRNA expression level compared to an average of those in normal kidneys of three control rats.

Chromosome 5 underwent an extensive loss in copy number, not less than that in other chromosomes (e.g., chromosomes 6, 8 and 20) (**Fig. 1B**). As it relates to extensive loss, homozygous deletions were most frequently observed at the *Cdkn2a/2b* locus on chromosome 5 (**Fig. 2A**). This commonly deleted region included two loci (*Cdkn2a* and *Cdkn2b*) for three distinct tumor suppressor genes (*p16* and *p19* in *Cdkn2a*; *p15* in *Cdkn2b*) (**Fig. 2B**). Shutdown of *p16/p19* and *p15* mRNA expression was confirmed in the samples that contained a homozygous deletion at the *Cdkn2a/2b* locus (**Figs. 2C and 2D**). In samples with a hemizygous deletion at the *Cdkn2a* locus (i.e., FB32-4, FB28-7), *p16* and *p19* expressions were downregulated, presumably because the promoter regions of the remaining alleles were methylated. However, some of the samples with either a hemizygous or no deletion (e.g., FB14-3; BF51-1; FB14-6; FB30-5 and FB33-7) showed a marked overexpression of *p16* and *p19*.

### Frequent Amplifications Over the Pericentromeric Region of Chromosome 4 and Amplification at the *Met* Locus

**Figure 3:**
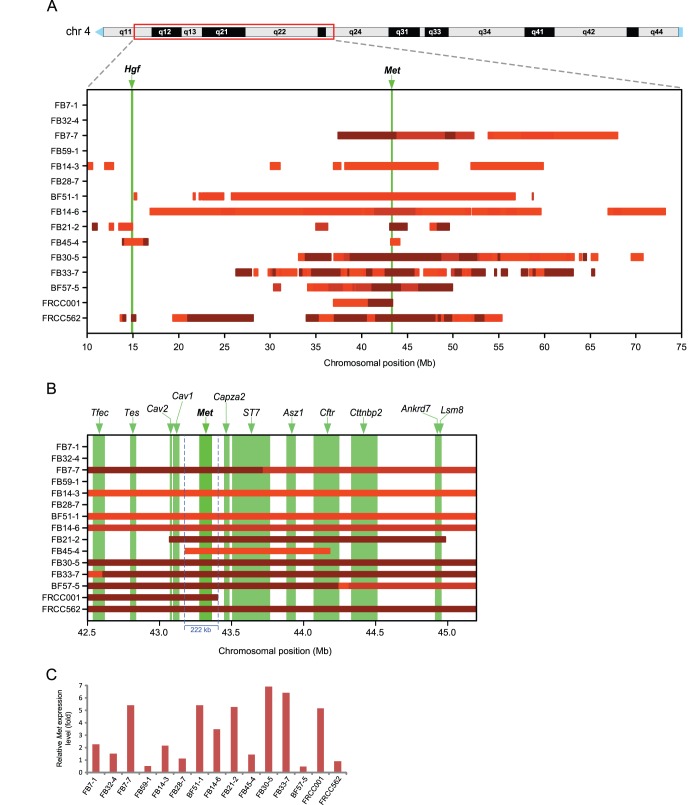
Frequent wide-ranging amplifications over a long pericentromeric region of chromosome 4 with the *Met* oncogene residing in the most overlapping section. (A) The bar chart represents the amplification regions along a 65 Mb pericentrometic region of chromosome 4 for 13 RCC tumors and two RCC cell lines. Four grades of amplification are indicated by bar color gradation; the darker the red, the larger the amplitude. (B) A magnified view of the bar chart above shows the vicinity of the most overlapping region. The genomic regions of all of the RefSeq genes included in the displayed range of the chromosome are depicted as vertical bars in the background. (C) Expression analysis of *Met* for 13 RCC tumors and two RCC cell lines by real-time PCR. The values on the *y*-axis indicate relative mRNA expression level compared to an average of those in normal kidneys of three control rats.

Over a long portion of the pericentromeric region in chromosome 4, frequent copy number gain and amplification were observed (**Fig. 1B**). Genomic amplification and gene expression in the corresponding areas of chromosome 4 were mostly proportional (**Fig. S3**). A bar plot of the amplified region along the pericentromeric region in chromosome 4 revealed that *Met* oncogene resides in the most common overlapping genomic section (**Fig. 3A**). The most overlapping section with a length of approximately 222 kb consisted of an amplified region in 11/15 samples and contained only one RefSeq-curated gene (*Met*) (**Fig. 3B**). A greater than 5-fold increase in *Met* mRNA expression was observed in 6 samples among the 9 tumors that contained a genomic amplification of *Met* (**Fig. 3C**).

Collectively, regarding chromosomal aberrations at these two cancer-related loci, every examined carcinoma including the two cell lines contained either the *Met* amplification or the *Cdkn2a/2b* deletion (**Table 1** **,** **Figs. 2B** **and** **3B**). Other common genetic alterations are summarized in **Table S1** (20 deleted genes, common in 2–4 RCC tumors) and **Table S2** (340 amplified genes, common in 2–9 RCC tumors). Among those *Zbtb38* amplification was confirmed for overexpression (**Fig. S4**).

**Table 1:** Features of 13 cases of Fe-NTA-induced renal cell carcinomas.

						Copy number aberration at	
Tumor case	Size (mm)	Metastasis	Invasion	Nuclear atypia grade	Growth pattern	*Met* locus	*Cdkn2a/2b* locus
FB7-1	20	None	None	Low	Intermediate	None	Loss
FB32-4	15	Lung	None	Intermediate	Intermediate	None	Loss
FB7-7	60	Lung	None	Intermediate	Expansive	Amplification	None
FB59-1	15	Lung	Peritoneal	Intermediate	Infiltrating	None	HD
FB14-3	15	None	None	High	Expansive	Amplification	Loss
FB28-7	30	None	None	High	Intermediate	None	Loss
BF51-1	28	None	None	High	Intermediate	Amplification	None
FB14-6	30	Lung	Peritoneal	High	Intermediate	Amplification	Loss
FB21-2	40	None	None	High	Infiltrating	Amplification	HD
FB45-4	40	Lung	None	High	Infiltrating	Amplification	HD
FB30-5	60	Lung	Peritoneal	High	Infiltrating	Amplification	Loss
FB33-7	70	Lung	Peritoneal	High	Infiltrating	Amplification	None
BF57-5	25	Lung	Peritoneal	High	Infiltrating	Amplification	HD

Fe-NTA: ferric nitrilotriacetate; HD: homozygous deletion.

### Tumor Size of Fe-NTA-induced RCCs is Regulated by Genetic Features

**Figure 4:**
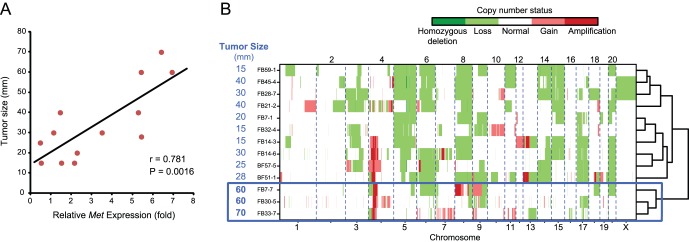
Tumor sizes of Fe-NTA induced RCCs are controlled by the genetic features. (A) *Met* expression is significantly correlated with tumor size. Pearson’s correlation coefficient (r) and the corresponding P value are written on the plot area. (B) Hierarchical clustering of the RCC tumors based on the whole genome patterns of the copy number changes. The large-size tumors form a distinct cluster.

We examined the associations between genetic alterations and various RCC traits including those summarized in **Table 1**. Among all of the relationships examined, *Met* expression and tumor size were proportionally associated (**Fig. 4A**). Furthermore, a hierarchical clustering of tumors based on whole patterns of chromosomal changes revealed that a group of large tumors (i.e., FB7-7, FB30-5 and FB33-7) corresponded to a distinct cluster (**Fig. 4B**). Therefore, a tumor trait, size at the time the tumor was clinically overt, was associated with the entire array-based CGH profiles.

### Comparison of Copy Number Alteration Profiles in Cancer Genomes Between Rats and Humans

**Figure 5:**
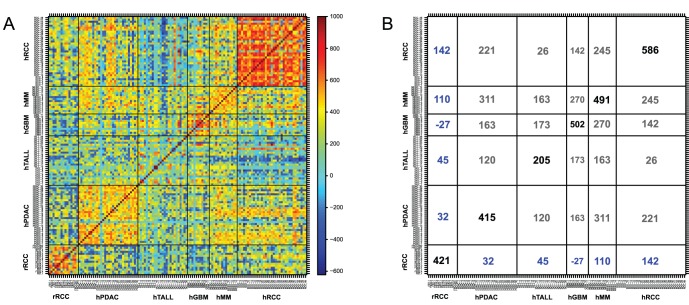
Comparison of copy number alteration profiles in cancer genomes between Fe-NTA-induced rat RCCs and human tumors. (A) The color plot represents a similarity matrix across the rat RCCs and various human cancers. rRCC, rat renal cell carcinoma; hPDAC, human pancreatic ductal adenocarcinoma; hTALL, human T-cell acute lymphoblastic leukemia; hGBM, human glioblastoma multiforme; hRCC, human renal cell carcinoma. (B) Numerical summary of the similarity matrix. The number in each square indicates an average value of similarity index (defined between −1000 and 1,000). Refer to the Materials and Methods section for details.

To determine the general principle of large-scale genomic changes in cancer across mammalian species, we compared cancer genomes of rats and humans as a whole spectrum of chromosomal alterations. First, we transformed the rat array-based CGH profiles onto human chromosomes according to a synteny between the two species. Thereafter, we compared the whole patterns based on estimated copy numbers using multidimensional data analysis methods. We found that Fe-NTA-induced rat RCC was most similar to human RCCs, followed by human malignant mesothelioma (**Figs. 5A and 5B**).

## Discussion

In this study, we report for the first time analyses of the entire data from array-based CGH applied to a Fenton chemistry-induced carcinogenesis in a rat kidney model [20]. We found that oxidative stress causes extensive genomic alterations in the induced cancer genome at chromosomal level (**Fig. 1A**). It is well known that a majority of human malignant solid tumors possess gains or losses in numerous chromosomes [21], [22], and amplification can be a suitable target for cancer chemotherapy. During the carcinogenic process of such tumors, chromosomal instability is thought to contribute as a driving factor [23]. Among wild-type rodent carcinogenesis models, however, few models report using primary tumor samples extensive genetic alterations because of chromosomal instability [24], [25]. Radiation-induced murine malignant lymphoma [26] and murine lung adenocarcinoma induced by 4-(methylnitrosamino)-1-(3-pyridyl)-1-butanone, a carcinogen present in tobacco smoke [27], revealed slightly more gross chromosomal aberrations than the corresponding spontaneous tumors, albeit the low resolution in the report (bacterial artificial chromosome [BAC] array of ∼6,500). The facts that control rats exhibit no RCCs [11], [12] and Fe-NTA-induced rat RCC model exhibits an equivalence to human cancers in genomic alterations at chromosomal level strongly support the idea that this carcinogenesis model mimics an actual carcinogenic process in those humans who lack strong cancer susceptibility traits.

Conversely, mice with multiple genetically-engineered cancer-associated genes show genetic alteration of this kind [28]. We think that those experiments correspond to an established mutator phenotype [29] and, thus, to the carcinogenic process in humans who have strong cancer susceptibility traits such as Li-Fraumeni syndrome (*p53*) [30] or melanoma kindreds (*p16*) [31]. As a hereditary rat RCC model, we also analyzed the RCCs of Eker rats, which do not show aggressive characteristics, such as metastasis [32], [33], [34]. We observed that these RCCs showed null or subtle genetic alterations (**Fig. S5**). Accordingly, oxidative stress, including that induced due to excess iron, could be one of the causes of human renal carcinogenesis. Indeed, numerous epidemiological studies have associated iron and steel industry workers with an increased RCC risk [35].

A frequency plot analysis revealed two remarkable features. First, the chromosomal aberrations showed a preference for loss against the ploidy of each cancer genome. Mostly, the aberration was represented by a deletion at either a whole chromosomal level (monosomy) or at a segmental level (**Fig. 1B**). The most common target for loss was the *Cdkn2a/2b* locus. The predominance of loss in the profile of chromosomal alterations may be attributed to the early stage of carcinogenesis. We previously demonstrated that cells with a hemizygous deletion at *Cdkn2a* appear as early as a few weeks after initiating a Fe-NTA administration [36].

Our present analysis revealed that the monoallelic loss of chromosome 5 in its entirety or of an equivalently wide region is the major first event. Indeed, we found only one case (6.7%) of a monoallelic loss of an extremely narrow region (∼350 kilobases; **Figs. 2A and 2B**). Fe-NTA catalyzes the generation of hydroxyl radicals through a Fenton reaction specifically in the lumina of renal proximal tubules, which leads to degeneration and necrosis/apoptosis of those cells [37], [38]. Because kidney is a vital organ that performs urea excretion, reabsorption of valuable molecules as well as ionic homeostasis maintenance, regeneration from the remaining tubular cells is intensely promoted. Under chronic oxidative stress by repeated Fe-NTA administrations, this degeneration and regeneration process would continue for months to years, increasing the risk of mitotic events simultaneously with the repair of oxidative DNA damage. We believe that this oxidative stress causes abnormal DNA replication and chromosomal missegregation, which leads to the emergence of aneuploid cells. Surprisingly, this series of events appear to occur in months, leading to a high incidence (∼90%) of RCC in rats within two years. Aneuploid cells usually exhibit phenotypes consistent with increased energy need and proteotoxic stress. However, aneuploidy can promote tumorigenesis under the following two hypothetical mechanisms: 1) aneuploidy may cause a proliferative advantage through loss of G1/S transition control under conditions in which normal euploid cells do not divide [39] and 2) aneuploidy can advance tumorigenesis by promoting genomic instability, hence increasing the evolvability of tumors [40]. The frequent deletion of the *Cdkn2a*/*2b* locus is observed in the rat peritoneal mesothelioma, an iron overload-associated tumor [41], induced either by another iron compound (ferric saccharate) [42] or by asbestos [43]. These common traits of animal models strongly suggest that *Cdkn2a*/*2b* is a principal target in iron-mediated carcinogenesis. The same genetic alteration is observed in rat mesothelioma induced by multi-walled carbon nanotubes [44], in which iron involvement is not yet established. We would like to add here for the biological significance of our results that homozygous deletion of *CDKN2A/2B* is frequently observed in human mesothelioma associated with asbestos exposure [**45**], [**46**].

Some of the tumors with a remaining *Cdkn2a*/*2b* allele showed extremely high expression levels of two products from this locus, *p16/Ink4a* and *p19/Arf.* This is a currently debated issue in human cancer [47]. There is considerable evidence that several neoplasms exhibit significant p16 levels in cytoplasm [48]. This can be an unsuccessful attempt to stop cell proliferation in the case of downstream *Rb* dysregulation [49] or may represent an alternative mechanism for modulating unidentified pathways. Our data exhibit ∼50% hemizygous deletion of *Rb*. This requires further clarification with epigenetic analysis.

The second feature determined using frequency plot analysis was a high incidence of amplification along a limited chromosomal region toward the centromere of chromosome 4, pointing to the *Met* locus. A region spanning 80 Mb from the centromere of rat chromosome 4 is syntenic to human chromosome 7. Various human cancers, such as glioblastoma [50] and non-small cell lung cancer [51], are reported to harbor amplifications in chromosome 7. Tyrosine kinase MET is a receptor for a hepatocyte growth factor and is situated upstream of *ras* in the signal transduction pathway, thus serving as an advantage for cell proliferation [52]. Therefore, it is conceivable that tumor size was proportionally associated with the *Met* expression level (**Figs. 3C** **and** **4A**). Because we dissect the animal as soon as we recognize the tumor, we believe that large-sized tumors are more aggressive in nature. It is of note that tumor size was also related to the genome alteration pattern (**Fig. 4B**), and was associated with amplification and overexpression of *Zbtb38* located on chromosome 8 (**Fig. S4**). ZBTB38 (CIBZ) represses the transcription of methylated templates [53], thus presumably regulating epigenetic mechanisms. Down-regulation of ZBTB38, a novel substrate of caspase-3, induces apoptosis [54] and this gene is localized in a prostate cancer susceptibility locus [54]. These results confirm the possibility of tumor classification using array-based CGH**.** *Met* arose evolutionally late and is unique to mammals [52]; it could thereby be associated with the unique amplification in the whole genome.

Genomic amplification is hypothesized to occur via the breakage-fusion-bridge cycle [55], [56], [57]. A Fenton reaction causes double-stranded DNA breakage [10]. Our results revealed that these amplifications consisted of a mixture of wide-range low-level amplifications and fragmented, narrow high-level amplifications (**Fig. 3A**). This suggests a mechanism of positive feedback for amplification, starting from wide-range low-level amplification. We suspect an involvement of double-minutes, and a presence of susceptible genomic loci. This hypothesis requires further study. It was interesting that two tumor suppressive genes, *Cav1* [58], [59] and *ST7* [60], surrounded the *Met* locus (**Fig. 3B**). This may be the reason why the *Met* locus was a denominator for the rat RCCs. Whole exome or genome sequencing may further reveal new findings regarding point mutation and chromosomal translocation.

Finally, we compared the present rat results with corresponding human tumors by transforming data based on chromosomal synteny (**Figs. 5A and 5B**). It was expected that the genomic alteration of Fe-NTA-induced rat RCC was most similar to human RCC presumably because target cells are the same. However, surprisingly, human mesothelioma was the second most similar. It is now established that most human mesothelioma results from exposure to asbestos, and the primary pathogenic process involved is iron overload [**8]**, [**61**]. The same mesodermal origin of renal tubular cells and mesothelial cells may cause the similarity of the array-based CGH profiles. Endodermal tumor, such as pancreatic ductal adenocarcinoma (PDAC), and ectodermal tumor, such as glioblastoma multiforme (GBM), exhibited a significant difference in genomic profiles.

In conclusion, we showed that repeated Fenton reactions in wild-type rats induced cancer that recapitulated genomic alterations similar to those in human cancers, suggesting the involvement of oxidative stress as a major factor in human carcinogenesis. In this renal carcinogenesis model, preferred alterations were deletion; *Cdkn2A/2B* deletion and *Met* amplification were the major target gene modifications. A comparative interspecies analysis would contribute to identifying candidate carcinogenic agents.

## Materials and Methods

### Fe-NTA-induced Renal Cell Carcinoma Model

Fe-NTA-induced carcinogenesis experiments were performed using male F1 hybrid rats that were a cross between Fischer 344 and Brown-Norway inbred strains (Charles River, Yokohama, Japan) as previously described [62]. Thirteen RCC cases were used in this study, and the histological grade of the tumor was determined according to the modified World Health Organization classification as we previously described [62]. The details are summarized in **Table 1**. The animal experiment committees of the Graduate School of Medicine, Kyoto University Graduate School of Medicine and Nagoya University Graduate School of Medicine approved this study. FRCC001 and FRCC562 cell lines were established from primary Fe-NTA-induced RCCs as previously described [63].

### Array-based Comparative Genomic Hybridization

Genomic DNA from the tumors and the cell lines was isolated with DNeasy (Qiagen, Valencia, CA). Array-based CGH was performed with an Agilent 185 K rat genome CGH microarray (Agilent Technologies, Santa Clara, CA) as previously described [64]. Thirteen primary tumors and two cell lines of Fe-NTA induced RCCs were analyzed using reference DNA extracted from a normal kidney of a rat from Brown-Norway imbred strain. One RCC sample of a female Eker rat [32] was analyzed using reference DNA extracted from a normal kidney of a male Eker rat. Two additional RCC samples of male Eker rats were analyzed with Rat Genome CGH Microarray 105A (G4436A; Agilent Technologies), using reference DNA extracted from a normal liver of another male Eker rat. The normalized array-based CGH data were processed to generate a segmented profile by circular binary segmentation (CBS) [65] with an altered significance level (alpha = 0.0001). The procedure of data processing for copy number estimation is detailed in **Methods S1**.

### Quantitative RT-PCR Analysis

Total RNA was isolated using Isogen reagents (Nippon Gene Co. Ltd., Tokyo, Japan) according to the manufacturer’s protocol. cDNA was synthesized using an RNA PCR kit ver. 3.0 (Takara Bio, Shiga, Japan) with random primers. A Platinum SYBR Green qPCR SuperMix-UDG kit (Invitrogen, Carlsbad, CA) and a 7300 Real-Time PCR System (Applied Biosystems, Foster City, CA) were used for quantitative real-time PCR analysis. Rat β-actin was used as an internal control. The primers used were as follows: *p16^Ink4a^*, 5′-aaacgccccgaacactttc-3′ and 5′-gttcgaatctgcaccatagga-3′; *p19^Arf^*, 5′-accccaagtgagggttttct-3′ and 5′-agagctgccactttgacgtt-3′; *p15^Ink4b^*, 5′-tccacaggctagagggaaaa-3′ and 5′-gtgcaggtgactccttggtt-3′; *Met*, 5′-ttaagcgagagcacgacaaat-3′ and 5′-ccacataggaaaacgcactgt-3′; *Zbtb38*, 5′-gtagctgctgctccaaatcc-3′ and 5′-cctgttgagggtggtgaact-3′; β-actin, 5′-tgtgttgtccctgtatgcctctg-3′ and 5′-atagatgggcacagtgtgggtg-3′.

### Human Data

We used human array-based CGH data of pancreatic ductal adenocarcinoma (hPDAC) [28], T-cell acute lymphoblastic leukemia (hTALL) [28], glioblastoma (hGBM) [66], mesothelioma cell line (hMM) [67] and renal cell carcinoma (hRCC) [56]. The Agilent CGH-array data of the former four types of human cancer were obtained from NCBI’s Gene Expression Omnibus (GEO) website (http://www.ncbi.nlm.nih.gov/geo/). The GEO accession numbers for the data sets are GSE7599 (hPDAC), GSE7603 (hTALL), GSE9177 (hGBM) and GSE22237 (hMM). The human RCC data was obtained through analyses with BAC microarrays (4,361 clones) [68]. We defined similarity index between the two array-based CGH profiles as follows. First, we calculated the correlation coefficient with log2 ratios of the estimated copy number over the inferred cancer ploidy for the genomic positions corresponding to all of the Agilent 44 K human CGH microarray probes. Then, we multiplied the value by 1×10^3^ after changing the absolute value into its square root.

## Supporting Information

Figure S1 **Array-CGH profiles from all the RCCs examined.** Red lines show log2 ratios of estimated copy number over inferred cancer ploidy versus genomic position for all the CGH microarray probes.
(PDF)

Figure S2 **Distribution of log2 ratio values of estimated copy number for all the probes in all the microarrays performed.**
(PDF)

Figure S3 **Example of global expression changes in line with genomic alteration.** Differences in genome and transcriptome are analyzed between two RCCs, FB7-7 having wide-range amplification on chromosome 4 versus FB28-7 having no substantial genomic alteration on chromosome 4. (A) Sky blue circle plot indicates ratio of estimated copy numbers based on array-based CGH (FB7-7 *vs* FB28-7). Red circle plot indicates ratio of normalized signals on Affymetrix expression microarray (FB7-7 *vs* FB28-7). (B) Expression ratio values are averaged along the chromosome. Here, red circle plot indicates average value in 2-Mb windows.
(PDF)

Figure S4 ***Zbtb38*** **mRNA expression is demonstrably associated with its chromosomal copy number.** (A) Array-CGH profiles of two RCC tumors harboring amplification over *Zbtb38* locus on chromosome 8. (B) Expression analysis of *Zbtb38* on 13 RCC tumors by real-time PCR. The values of the *y*-axis indicate the relative mRNA expression level compared to an average of those in normal kidneys of three control rats.
(PDF)

Figure S5 **Array-CGH profiles of three hereditary (Eker rat) renal tumors.** Red lines show log2 ratios of estimated copy number over inferred cancer ploidy versus genomic position for all the CGH microarray probes.
(PDF)

Table S1 **List of genes completely deleted in more than two RCC tumors.**
(XLS)

Table S2 **List of genes amplified in more than two RCC tumors.**
(XLS)

Methods S1 **Supplementary methods.**
(DOC)
