# Supplementary figures and images for: Fenton Reaction Induced Cancer in Wild Type Rats Recapitulates Genomic Alterations Observed in Human Cancer
Source: PLoS One. 2012 Aug 29;7(8):e43403. doi: 10.1371/journal.pone.0043403 (PMC3430702; doi:10.1371/journal.pone.0043403)

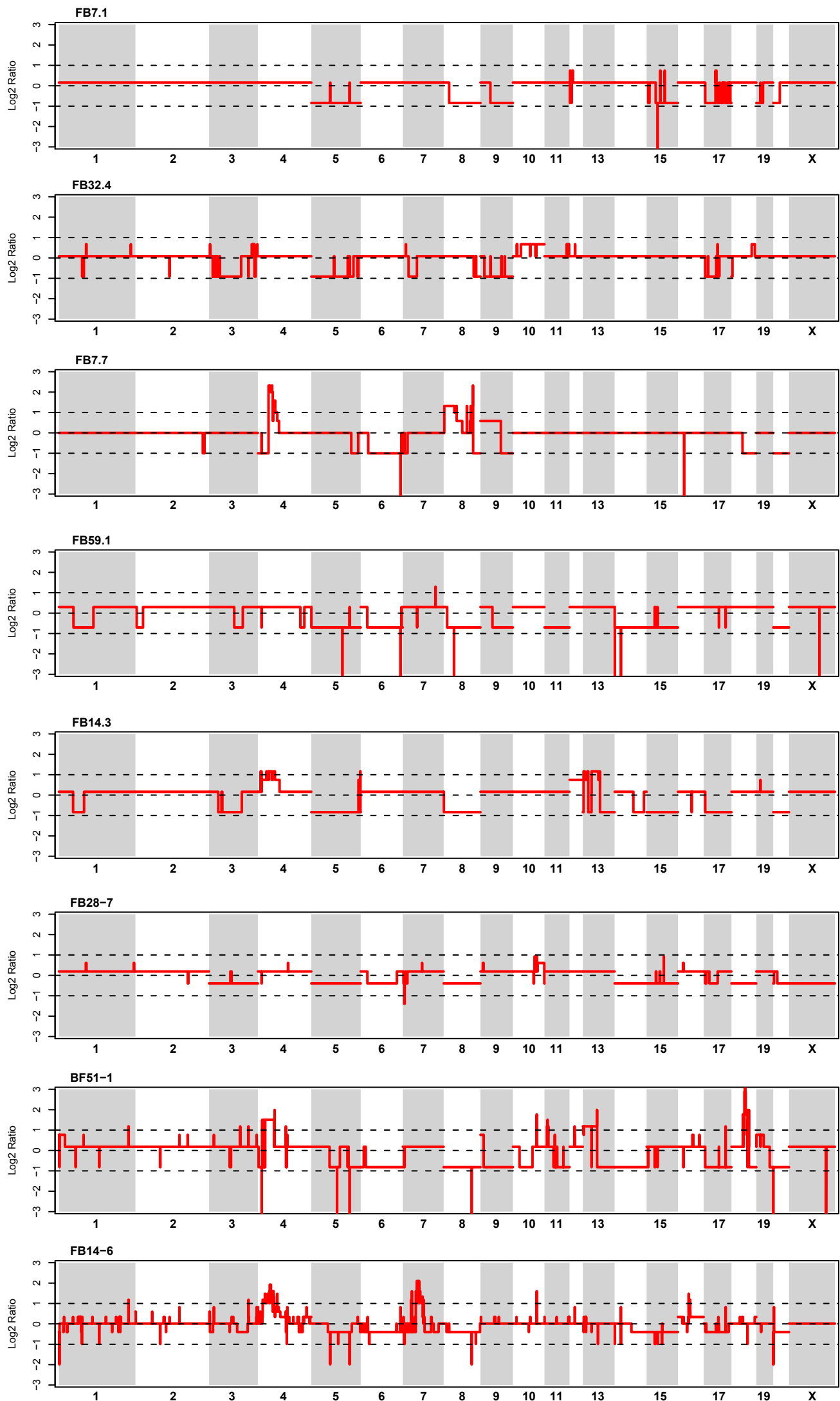

Figure S1

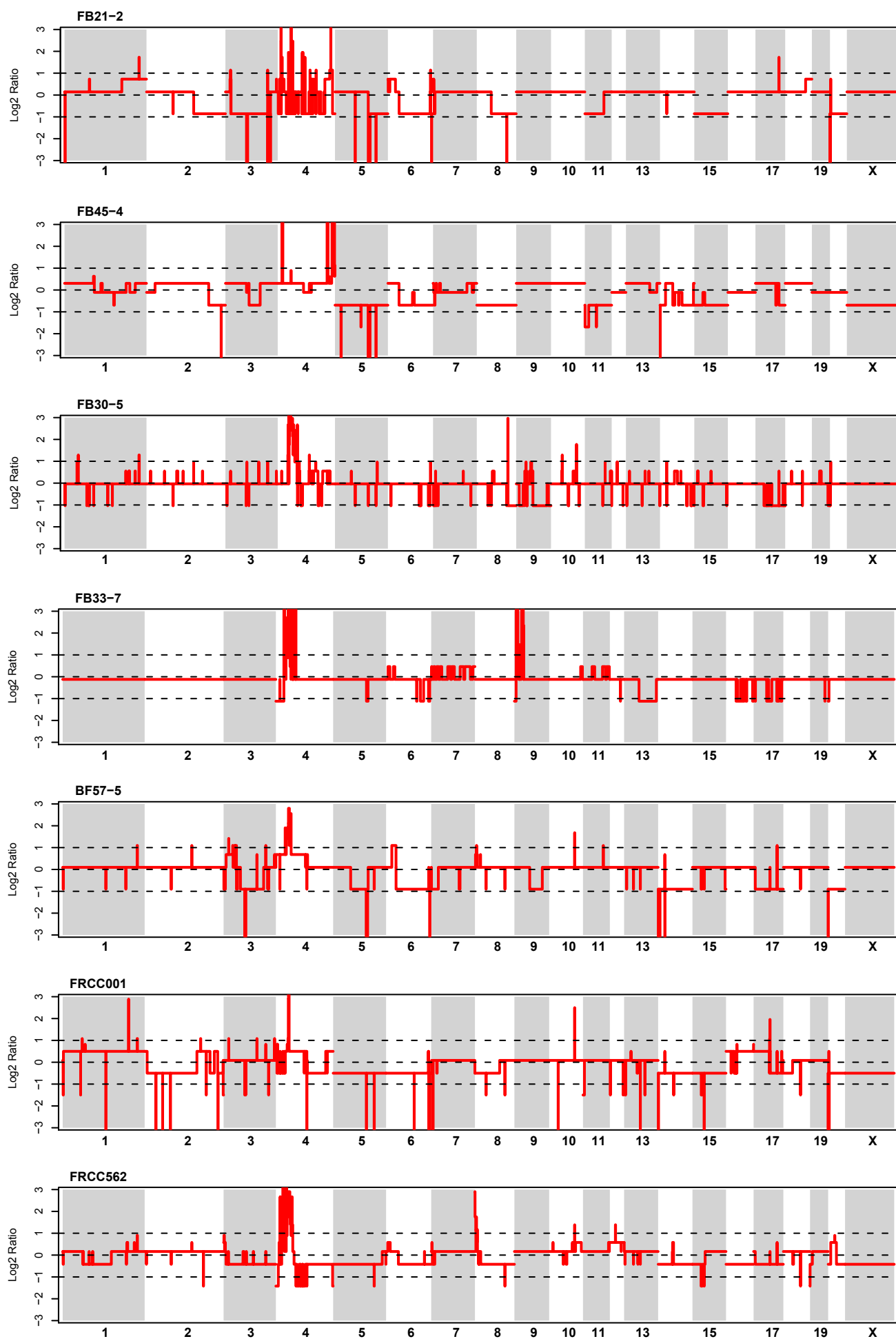

Figure S1

Supplement: Figure S1 — Array-CGH profiles from all the RCCs examined. Red lines show log2 ratios of estimated copy number over inferred cancer ploidy versus genomic position for all the CGH microarray probes. (PDF) [file pone.0043403.s001.pdf]

# Histogram of recalculated log2Ratio values for all samples (x all probes)

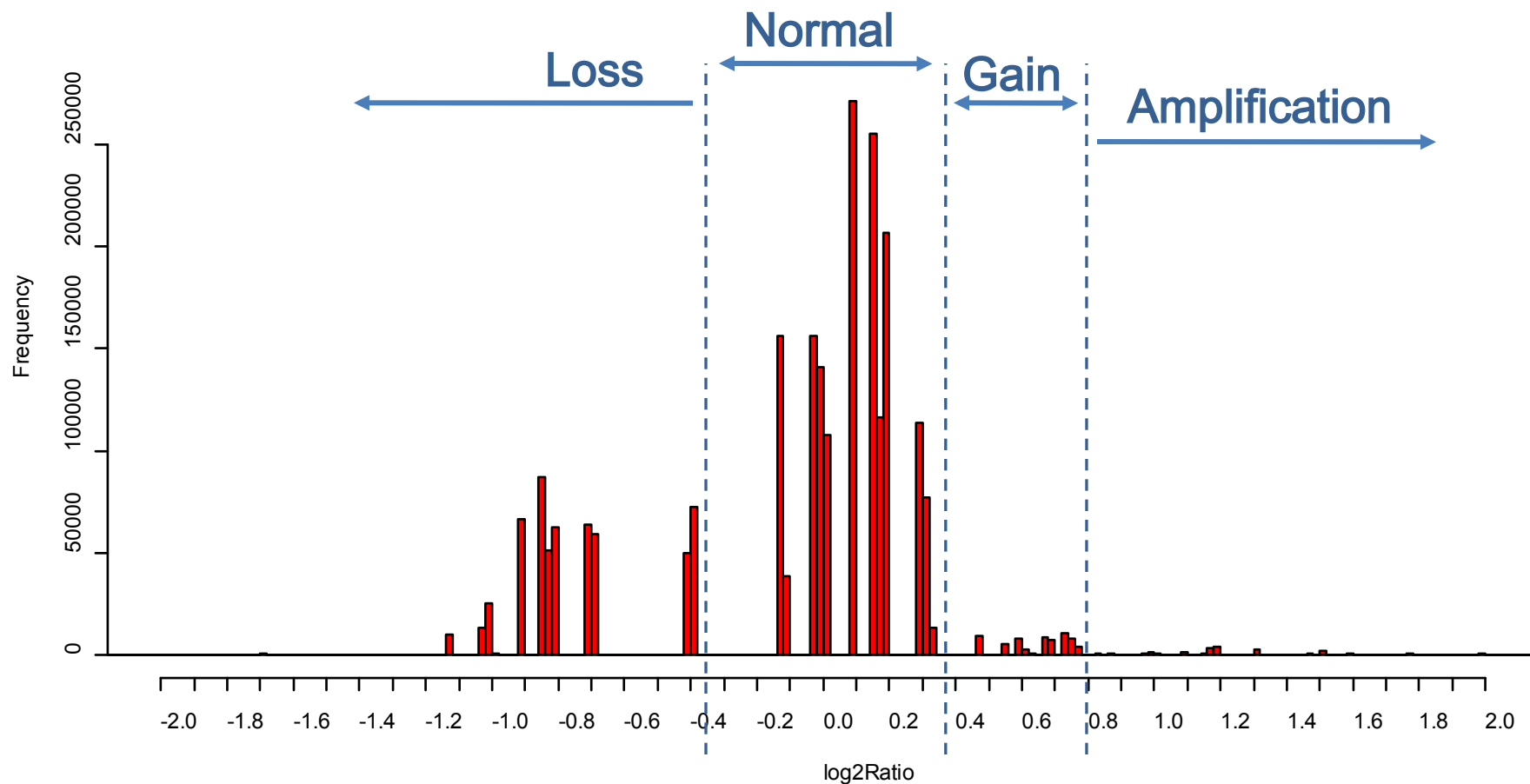

Figure S2

Supplement: Figure S2 — Distribution of log2 ratio values of estimated copy number for all the probes in all the microarrays performed. (PDF) [file pone.0043403.s002.pdf]

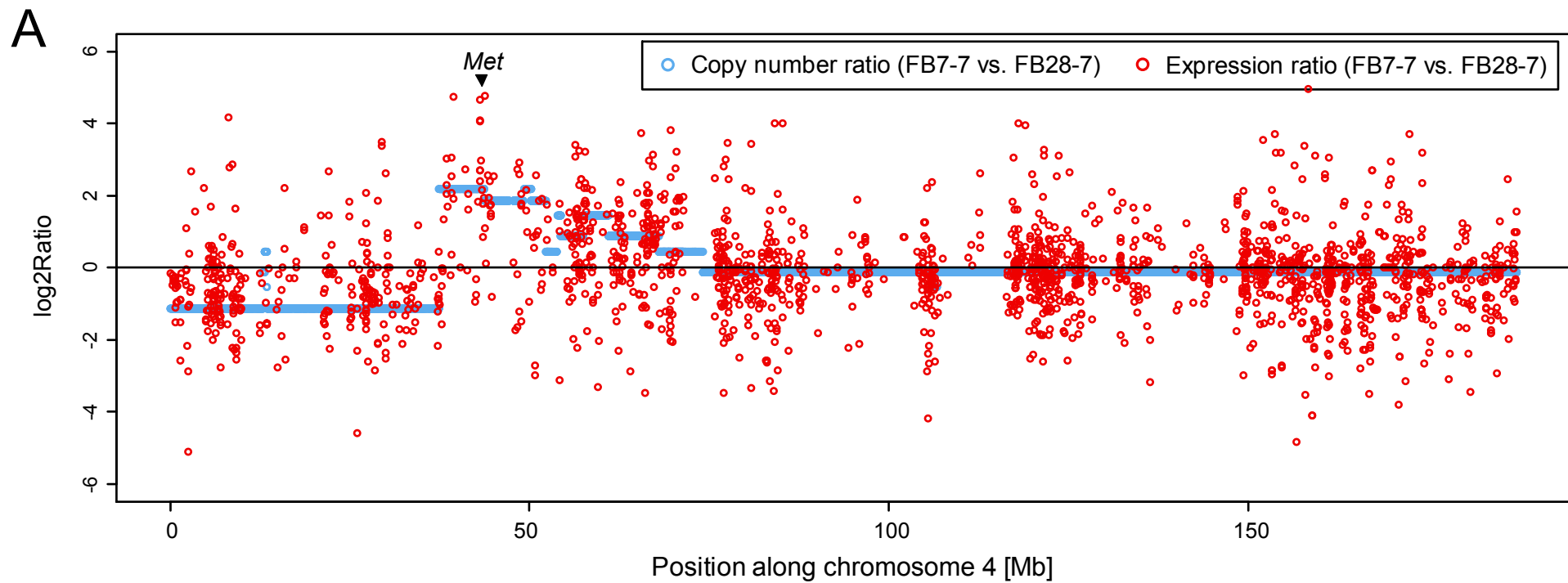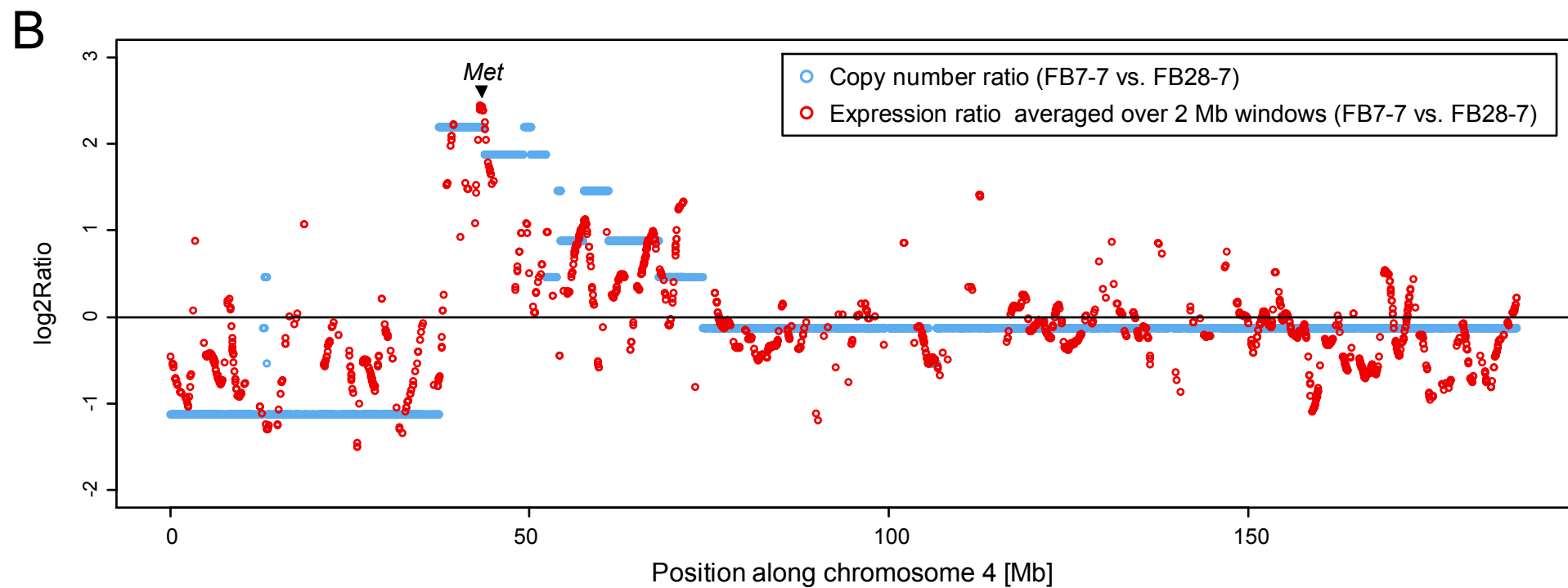

Supplement: Figure S3 — Example of global expression changes in line with genomic alteration. Differences in genome and transcriptome are analyzed between two RCCs, FB7-7 having wide-range amplification on chromosome 4 versus FB28-7 having no substantial genomic alteration on chromosome 4. (A) Sky blue circle plot indicates ratio of estimated copy numbers based on array-based CGH (FB7-7 vs FB28-7). Red circle plot indicates ratio of normalized signals on Affymetrix expression microarray (FB7-7 vs FB28-7). (B) Expression ratio values are averaged along the chromosome. Here, red circle plot indicates average value in 2-Mb windows. (PDF) [file pone.0043403.s003.pdf]

# A

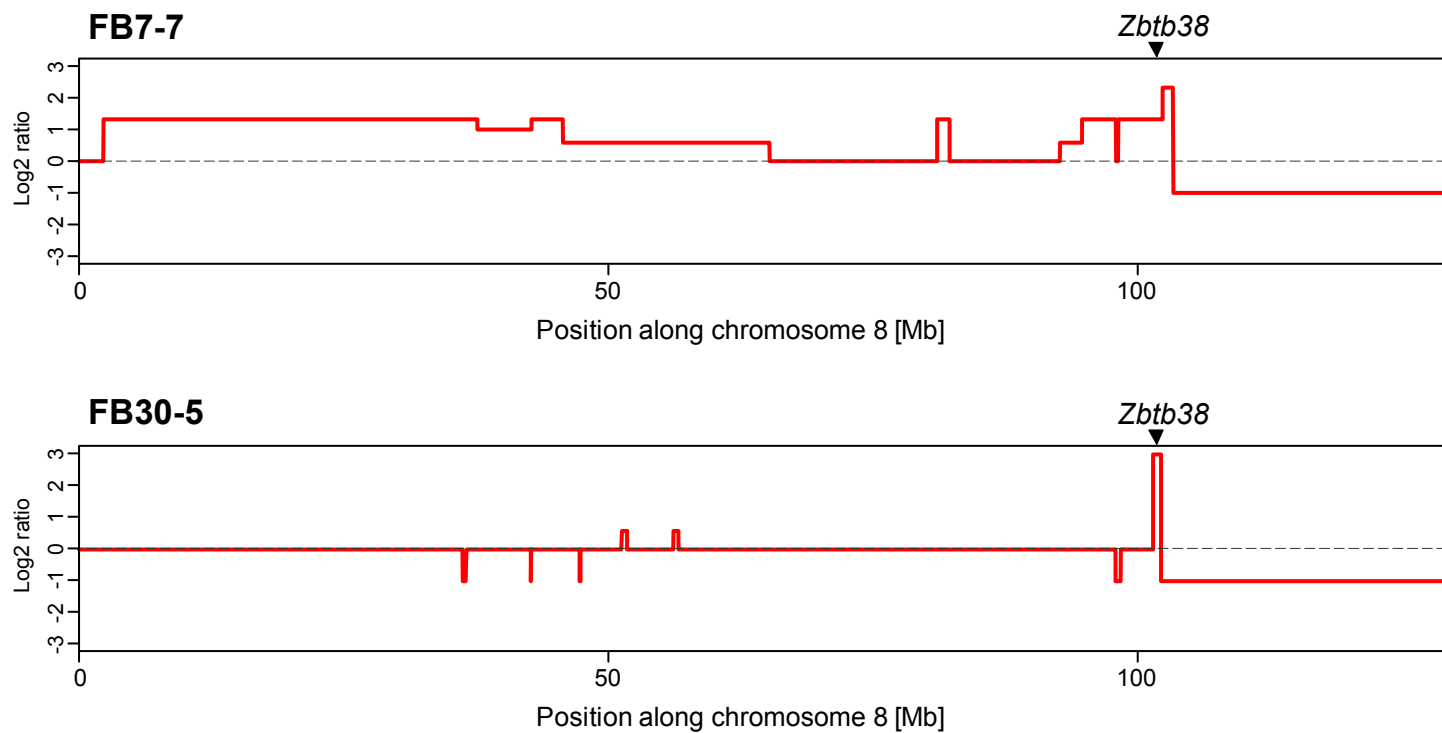

# B

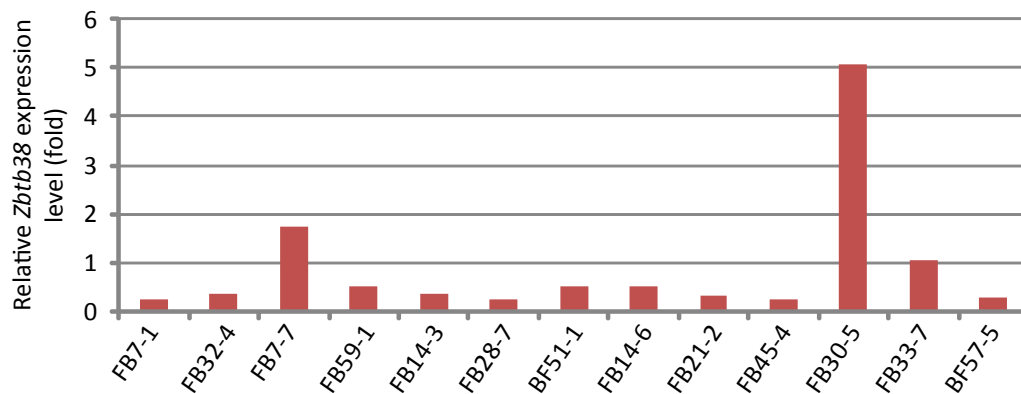

Supplement: Figure S4 — Zbtb38 mRNA expression is demonstrably associated with its chromosomal copy number. (A) Array-CGH profiles of two RCC tumors harboring amplification over Zbtb38 locus on chromosome 8. (B) Expression analysis of Zbtb38 on 13 RCC tumors by real-time PCR. The values of the y-axis indicate the relative mRNA expression level compared to an average of those in normal kidneys of three control rats. (PDF) [file pone.0043403.s004.pdf]

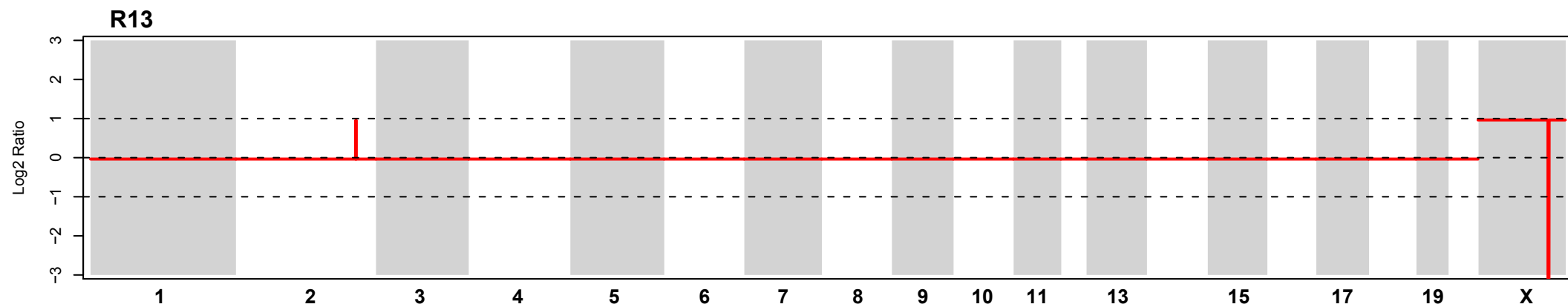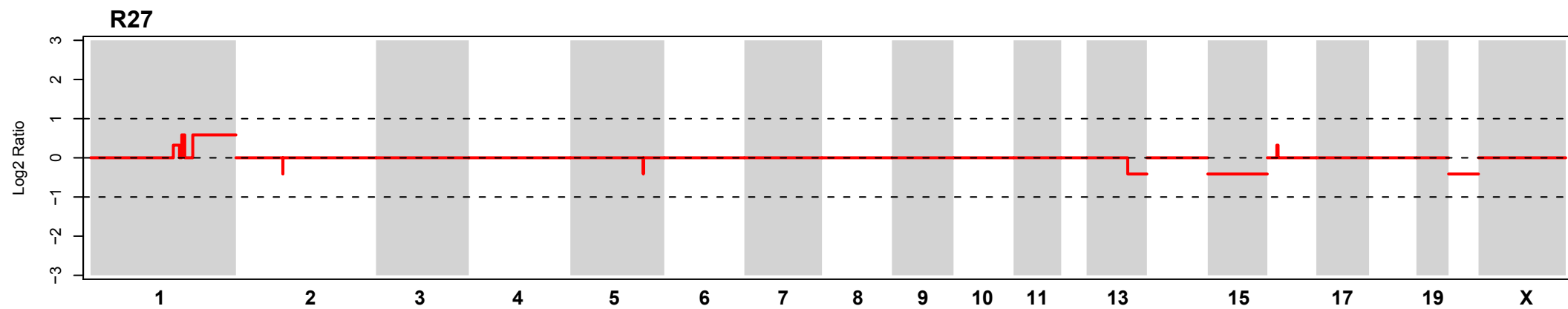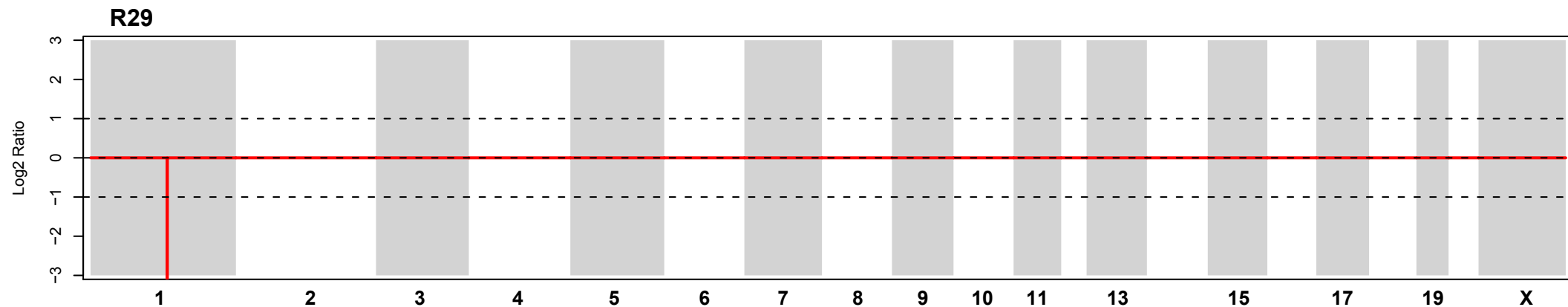

Figure S5

Supplement: Figure S5 — Array-CGH profiles of three hereditary (Eker rat) renal tumors. Red lines show log2 ratios of estimated copy number over inferred cancer ploidy versus genomic position for all the CGH microarray probes. (PDF) [file pone.0043403.s005.pdf]
