# Supplementary material for: Fenton Reaction Induced Cancer in Wild Type Rats Recapitulates Genomic Alterations Observed in Human Cancer
Source: PLoS One. 2012 Aug 29;7(8):e43403. doi: 10.1371/journal.pone.0043403 (PMC3430702; doi:10.1371/journal.pone.0043403)
Supplement: Methods S1 — Supplementary methods. (DOC) [file pone.0043403.s008.doc]

**Supplementary Methods**

**Hybridization condition for each microarray**

Sample combinations for array-based CGH are summarized in the table below.

| **Sample for test DNA** | | | | **Sample for reference DNA** | | |
| --- | --- | --- | --- | --- | --- | --- |
| **Name** | **Animal** | **Sex** | **Cell type** | **Animal** | **Sex** | **Cell type** |
| FB7-1 | F1 hybrid | Male | Fe-NTA induced RCC tumor | Brown-Norway | Male | Normal kidney |
| FB32-4 | F1 hybrid | Male | Fe-NTA induced RCC tumor | Brown-Norway | Male | Normal kidney |
| FB7-7 | F1 hybrid | Male | Fe-NTA induced RCC tumor | Brown-Norway | Male | Normal kidney |
| FB59-1 | F1 hybrid | Male | Fe-NTA induced RCC tumor | Brown-Norway | Male | Normal kidney |
| FB14-3 | F1 hybrid | Male | Fe-NTA induced RCC tumor | Brown-Norway | Male | Normal kidney |
| FB28-7 | F1 hybrid | Male | Fe-NTA induced RCC tumor | Brown-Norway | Male | Normal kidney |
| BF51-1 | F1 hybrid | Male | Fe-NTA induced RCC tumor | Brown-Norway | Male | Normal kidney |
| FB14-6 | F1 hybrid | Male | Fe-NTA induced RCC tumor | Brown-Norway | Male | Normal kidney |
| FB21-2 | F1 hybrid | Male | Fe-NTA induced RCC tumor | Brown-Norway | Male | Normal kidney |
| FB45-4 | F1 hybrid | Male | Fe-NTA induced RCC tumor | Brown-Norway | Male | Normal kidney |
| FB30-5 | F1 hybrid | Male | Fe-NTA induced RCC tumor | Brown-Norway | Male | Normal kidney |
| FB33-7 | F1 hybrid | Male | Fe-NTA induced RCC tumor | Brown-Norway | Male | Normal kidney |
| BF57-5 | F1 hybrid | Male | Fe-NTA induced RCC tumor | Brown-Norway | Male | Normal kidney |
| FRCC001 | Wistar | Male | Fe-NTA induced RCC cell line | Brown-Norway | Male | Normal kidney |
| FRCC562 | Wistar | Male | Fe-NTA induced RCC cell line | Brown-Norway | Male | Normal kidney |
| R13 | Eker | Female | Hereditary RCC | Eker | Male | Normal kidney |
| R27 | Eker | Male | Hereditary RCC | Eker | Male | Normal liver |
| R29 | Eker | Male | Hereditary RCC | Eker | Male | Normal liver |

**Calculation procedure for copy number estimation**

At first, relate the processed signal ratio values to theoretical expressions by the following steps (1 ~ 2), taking two factors causing the systematic deviation of the ratio values into account.

(1) Represent the signal ratio by an expression taking mean copy number over the whole genome of the tumor cells (ploidy) into account.

$$Signal ratio= \frac{ C^{T} }{ L^{T} }$$

*C^T^*: copy number at an individual genomic location in the tumor cells. *L^T^*: mean copy number over the whole genome of the tumor cells. This equation is valid when the copy numbers of the reference genome at each chromosomal location are assumed to be constant.

(2) Modify the above equation, additionally taking contamination with untargeted normal cells (proportion) into account.

$$Signal ratio = \frac{ C^{T}r+C^{N}(1-r) }{ L^{T}r+L^{N}(1-r) }$$

r: proportion of the targeted (tumor) cells in the cell population served for the extraction of test DNA. *C^N^*: copy number at the genomic location in the untargeted normal cells. *L^N^*: mean copy number over the whole genome of the untargeted normal cells. In this study, we adopted an assumption for the contaminated cells that the copy number of all the genomic locations equal 2. That is,

$$Signal ratio = \frac{ C^{T}r+2(1-r) }{ L^{T}r+2(1-r) }$$

Then, determine the optimal set of the two parameters (*L^T^* and *r*) as the sum of the errors from the nearest integer values is minimized for all the copy numbers calculated with the processed ratio values through the following steps (1 ~ 4). (1) For individual microarray probes, calculate the copy number by using the following equation with arbitrarily-specified values of *L^T^* and *r*.

$$Copy number=\frac{ Signal ratio \cdot\{L^{T}r+2(1-r)\} - 2(1-r) }{r}$$

(2) Calculate the difference between the calculated copy number and its nearest integer value to the probe as the residual error. (3) Sum up the squares of the residual errors for all the probes. (4) Search for the set of values of the parameters (*L^T^* and *r*) to minimize the sum of squares.

After determining the optimal values for the parameters (*L^T^* and *r*), copy number for each probes is calculated by assigning the values to the above equation. Finally, the real-valued copy number was rounded into the nearest integer value.

The optimal values of *L^T^* and *r* we obtained are shown below.

| Sample | *L^T^* (ploidy) | *r* (proportion) |
| --- | --- | --- |
| FB7-1 | 1.77 | 0.77 |
| FB32-4 | 1.8 | 0.844 |
| FB7-7 | 1.78 | 0.714 |
| FB59-1 | 1.65 | 0.704 |
| FB14-3 | 1.71 | 0.606 |
| FB28-7 | 2.45 | 0.792 |
| BF51-1 | 1.67 | 0.84 |
| FB14-6 | 3.76 | 0.632 |
| FB21-2 | 1.72 | 0.652 |
| FB45-4 | 2.74 | 0.668 |
| FB30-5 | 1.94 | 0.752 |
| FB33-7 | 1.91 | 0.746 |
| BF57-5 | 1.82 | 0.762 |
| FRCC001 | 2.56 | 0.84 |
| FRCC562 | 2.42 | 0.868 |

**Gene Expression Microarray**

Gene expression microarray was performed with the Rat Genome 230 2.0 array (Affymetrix Inc., Santa Clara, CA) as previously described (Gene Expression Omnibus accession number GSE7625) [1]. Probe intensities were assessed using GeneChip Operating Software (GCOS 1.0, Affymetrix). Expreesion profiles were analyzed using GeneSpring GX 10.02.2 software (Agilent Technologies).

**Reference**

[1] Liu Y-T, Shang D-G, Akatsuka S, Ohara H, Dutta KK, et al. (2007) Chronic oxidative stress causes amplification and overexpresson of *ptprz1* protein tyrosine phosphatase to activate β-catenin pathway. Am J Pathol 171: 1978-1988.
